# Impact of Climate Change on Indoor Air Quality: A Review

**DOI:** 10.3390/ijerph192315616

**Published:** 2022-11-24

**Authors:** Aya Mansouri, Wenjuan Wei, Jean-Marie Alessandrini, Corinne Mandin, Patrice Blondeau

**Affiliations:** 1 Scientific and Technical Centre for Building (CSTB), Health and Comfort Department, 84 Avenue Jean Jaurès, 77447 Marne-la-Vallée, France; 2 Laboratoire des Sciences de l’Ingénieur pour l’Environnement (LaSIE), UMR CNRS 7356, La Rochelle University, 17042 La Rochelle, France

**Keywords:** global warming, temperature, humidity, air pollutants, IAQ

## Abstract

Climate change can affect the indoor environment due to heat and mass transfers between indoor and outdoor environments. To mitigate climate change impacts and adapt buildings to the changing environment, changes in building characteristics and occupants’ behavior may occur. To characterize the effects of climate change on indoor air quality (IAQ), the present review focused on four aspects: (1) experimental and modeling studies that relate IAQ to future environmental conditions, (2) evolution of indoor and outdoor air concentrations in the coming years with regard to temperature rise, (3) climate change mitigation and adaptation actions in the building sector, and (4) evolution of human behavior in the context of climate change. In the indoor environment, experimental and modeling studies on indoor air pollutants highlighted a combined effect of temperature and relative humidity on pollutant emissions from indoor sources. Five IAQ models developed for future climate data were identified in the literature. In the outdoor environment, the increasing ambient temperature may lead directly or indirectly to changes in ozone, particle, nitrogen oxides, and volatile organic compound concentrations in some regions of the world depending on the assumptions made about temperature evolution, anthropogenic emissions, and regional regulation. Infiltration into buildings of outdoor air pollutants is governed by many factors, including temperature difference between indoors and outdoors, and might increase in the years to come during summer and decrease during other seasons. On the other hand, building codes in some countries require a higher airtightness for new and retrofitted buildings. The building adaptation actions include the reinforcement of insulation, implementation of new materials and smart building technologies, and a more systematic and possibly longer use of air conditioning systems in summer compared to nowadays. Moreover, warmer winters, springs, and autumns may induce an increasing duration of open windows in these seasons, while the use of air conditioning in summer may reduce the duration of open windows.

## 1. Introduction

The mean global surface temperature in 2020 was 1.02 °C warmer than the baseline 1951–1980 mean [1]. This temperature rise has several direct health effects on humans, such as risks of hyperthermia due to exposure to high temperatures, and indirect health effects due to bad air quality [2]. Vardoulakis et al. [2] indicated that the increase in temperature may lead to higher indoor concentrations of airborne pollutants causing higher risks of allergy, cancer, and endocrine disruption. 

Nazaroff [3] classified the factors influencing indoor air quality (IAQ) in response to climate change into three categories: (1) factors related to pollutants such as the transfer of outdoor pollutants into the indoor environment, the pollutant emission from indoor materials and products, and pollutant partitioning between the gas and the adsorbed/absorbed phases; (2) factors related to the building properties, such as insulation, materials, heating and cooling systems, and availability of indoor air cleaners; (3) factors related to occupants’ activities and behaviors.

Regarding pollutant-related factors, outdoor hygrothermal conditions can influence temperature and relative humidity indoors due to heat transfer through the building envelope. Changes in indoor thermal conditions can affect the mass transfer parameters, pollutant emissions from building materials, chemical reactivity, and pollutant partitioning between the gas and the adsorbed/absorbed phases (in materials, settled dust, airborne particles) [4]. Concerning building-related factors, energy issues and adaptation to climate change have already driven changes in the way of construction and designing heating, ventilation, and air conditioning (HVAC) systems in buildings [5]. These include changes in insulation materials, solar protection, and wider use of air conditioning systems to avoid overheating and promote thermal comfort, among others [5]. Yang et al. [6] found that the cooling demand would increase under future climate scenarios, and consequently the use of cooling systems. Outdoor-originated pollutants, such as polycyclic aromatic hydrocarbons (PAHs), benzene, and ozone, enter the indoor environment via the ventilation system, windows, and/or air infiltrations through cracks, as highlighted by Cheng et al. [7]. The indoor/outdoor pollutants transport can vary because of changes in building characteristics. The rise in outdoor temperatures may also change human behaviors. Weschler [8] showed that to handle hot weather, occupants of air-conditioned homes tended to operate their air conditioning systems and close windows rather than open windows and operate fans. 

To address the influencing factors of IAQ facing climate change, the present work aimed to conduct a literature review on the pollutant-, building-, and human-related factors, with a specific focus on modeling studies, which were never reviewed before. The review aimed to retrieve answers from the literature to the four following questions: (1) How did experimental and theoretical work characterize IAQ under future environmental conditions? (2) How would indoor and outdoor air concentrations evolve in the coming years with regard to climate change? (3) How may building characteristics evolve to mitigate the effects of climate change and adapt to the future climate, and how will these changes affect IAQ? (4) How may human behavior evolve in the context of climate change and influence IAQ? 

The impact on IAQ of extreme events generated by climate change, such as floods (development of mold in flooded buildings), hurricanes (carbon monoxide poisoning due to the temporary use of portable generators in the absence of electricity), or wildfires (huge increase in outdoor air particle concentrations) is also important [9] but is not addressed in this review. In a first attempt to model IAQ under global warming conditions, extreme events are not prioritized, as their predictions remain a challenge to date. 

## 2. Materials and Methods

**Figure 1:**
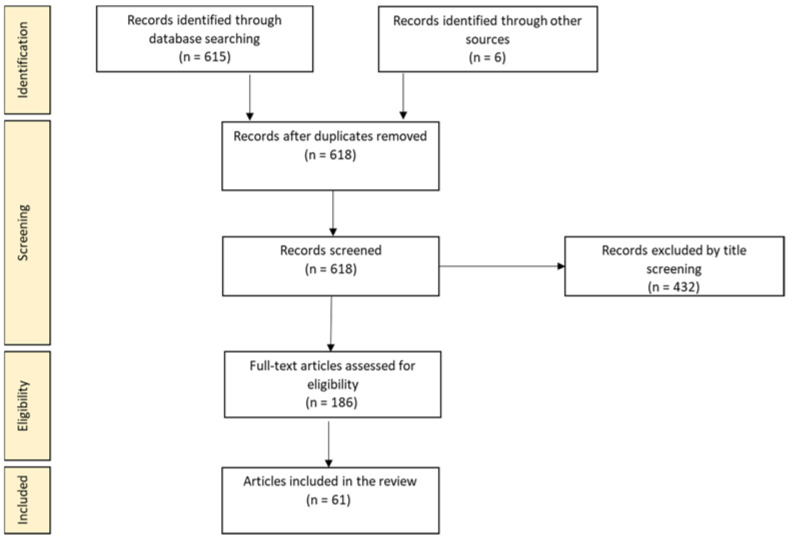
Review flow chart.

The literature review was conducted with ScienceDirect and Google Scholar search engines using the following keywords: (“climate change”) AND (“building”) AND ((“air temperature”) OR (“relative humidity”) OR (“volatile organic compound”) OR (“aldehyde”) OR (“particle”) OR (“SVOC”) OR (“indoor air quality”)), without year limitation. A total of 615 results were obtained. In addition, 6 papers identified from grey literature, including international guidelines and regulations for indoor air and health, were considered. After duplicate removal, 618 articles were screened, among which 432 were excluded by title screening because they were not relevant to the topic of the review. Finally, 186 articles were investigated in detail. After reading the full text, 61 articles were included in this paper (Figure 1).

**Figure 2:**
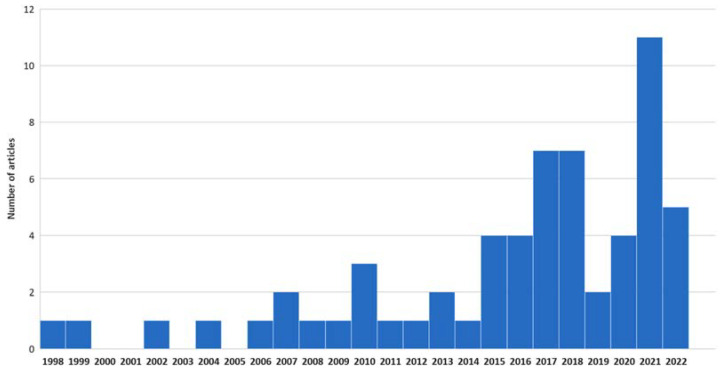
Evolution of the number of related articles with years.

The 61 selected articles were published between 1998 and 2022. Figure 2 shows the distribution of these articles over the total period. A growing interest can clearly be noticed. The studies were conducted in Australia, Chile, China, France, Germany, Japan, Malaysia, South Korea, Spain, Taiwan, the UK, and the US. Pollutants that were studied experimentally or numerically included volatile organic compounds (VOCs), semi-volatile organic compounds (SVOCs), ozone, nitrogen dioxide (NO_2_), radon, and airborne particles. Among the 61 articles, 15 addressed the experimental and theoretical work that characterized IAQ under future environmental conditions, 15 addressed the indoor/outdoor air concentration evolution with regard to climate change, 19 addressed the building characteristics evolution for climate change mitigation or adaptation, and 14 addressed the human behavior evolution in the context of climate change.

## 3. Results and Discussion

### 3.1. Models to Predict Indoor Pollutant Concentrations in the Context of Climate Change

Five models that were developed or used to predict IAQ under future climate conditions were identified. The corresponding equations and scope of application of each model are summarized in Table 1. A mechanistic model was developed by Chang et al. [10] to assess climate change impacts on indoor air concentrations of chemical pollutants. The model only applies to VOCs. It was used to predict the VOC concentrations in Korean houses for the period of 2011–2100. This model calculates indoor VOC concentrations with a 1 min time step based on inputs including the meteorological data, the room and window sizes, the outdoor VOC concentrations, the indoor VOC concentrations in the adjoining room, the building crack sizes, the air handling system, the recirculation filter characteristics, and the chemical properties of the target pollutants. Temperature-dependent parameters such as the discharge coefficient for window opening, the pollutant diffusion coefficients in indoor source materials, the air density, and the pollutant vapor pressure, as well as the effect of the indoor and outdoor temperatures on the window opening patterns and the heat transfer through the building envelope (and thus on indoor emissions and air transfer), were considered as the main inputs. The seasonal duration of open windows and the seasonal average of formaldehyde concentrations were predicted in South Korea for three periods, 30 years each, from 2011 to 2100, under three VOC emission scenarios: (1) no emission from indoor sources, (2) low and continuous emissions from vinyl flooring, and (3) high and periodic emissions to designate cooking activities. The first scenario is not realistic, but it allows us to identify the trend in concentration variations of pollutants originated outdoors. Occupants were not considered as a source in any of the three scenarios. The predicted formaldehyde concentrations showed a slight increase in the annual average concentration by approximately 12% in the 2071–2100 period due to the increase in its outdoor concentrations, assuming no emission from indoor sources or low emissions from vinyl flooring (scenarios 1 and 2). In the case of strong indoor emissions, the annual formaldehyde concentration would decrease by 3% due to pollutant removal by natural ventilation. On a seasonal scale, the simulation under the high and periodic emission scenario (scenario 3) showed that the average formaldehyde concentration would increase in summer and decrease in the other seasons, because of a lower air change and a higher air change in the future compared to buildings of nowadays. 

Ilacqua et al. [11] used a steady-state single-compartment model to predict the indoor air concentration of pollutants under future environmental conditions. The model only considers pollutant transports from outdoors to indoors by infiltrations through the building envelope. The infiltration in the paper of Ilacqua et al. [11] referred to the uncontrolled air flow through cracks and leaks in the building envelope and did not consider air change through mechanical ventilations or open windows. The infiltration rates were calculated based on the stack effect caused by the temperature difference between indoors and outdoors and the wind effect. The investigated pollutants were radon, PM_2.5_, ultrafine particles (UFP), carbonyls, ozone, NO_2_, and nitric acid (HNO_3_). The simulations did not consider any change in the building envelope’s airtightness in the future. The results suggested that the monthly mean infiltration rates in the 2040–2070 period would decrease in some American cities compared to the 1970–2000 period, except in summertime. Therefore, exposures to pollutants of outdoor origin would decrease while exposures to pollutants emitted by indoor sources would increase. The study showed that changes in buildings’ air infiltration in the future affected occupants’ pollutant exposure level. The decrease in infiltrations by 5% would increase the exposure to pollutants of indoor origin by 2 to 23% and decrease the exposure to pollutants of outdoor origin by 2 to 18%.

Salthammer et al. [12] developed a dynamic model for a single compartment to calculate indoor concentrations of ozone and particles in the 2040 in eight German cities located in different geographic zones. The model focuses on only outdoor sources and does not consider ozone and particle emissions from indoor sources. The indoor ozone concentration was calculated for a summer day with peak outdoor ozone concentrations. The diurnal indoor concentration did not exceed 100 µg·m^−3^, the WHO air quality guideline value [13], with an air exchange rate ranging from 0.5 to 3 h^−1^ depending on the period of the day. The model also predicted a decrease in indoor PM_2.5_ and PM_10_ concentrations, primarily due to lower outdoor particle concentrations in future emission scenarios.

In 2022, Salthammer et al. [14] developed a comprehensive modeling framework considering two compartments (gas and particulate phases) to better estimate the effect of climate change on IAQ. The target chemical pollutants were 12 VOCs and SVOCs: limonene, isoprene, formaldehyde, n-butyl acetate, n-decane, acetic acid, acetaldehyde, toluene, benzophenone, triethyl phosphate (TEP), 2,2,4-trimethyl-1,3-pentanediol diisobutyrate (TXIB), and di-1-ethylhexyl adipate (DEHA). The model includes five submodels to estimate the heat and moisture transport in buildings, indoor emissions, physicochemical processes, mold growth, and human exposure, respectively. The inputs of the model are the thermal boundary conditions, building materials, and occupants’ activities. The modeling framework has not been applied to future climates yet but the submodels were tested on existing data and past meteorological conditions. The thermal submodel was validated using indoor temperature and relative measured humidity in July and August 2020 in a house in Braunschweig, Germany. The outdoor temperature, relative humidity, and ozone concentration used in the simulations to validate the model were obtained from a monitoring station in the same city. The equations related to the chemical emissions and reactions were tested for limonene on 14 August 2020, and the emission rate of limonene was strongly and positively associated with indoor temperature. 

Fazli and Stephens [15] developed a dynamic single-compartment model to estimate indoor air concentrations of PM_2.5_, UFP, NO_2_, ozone, VOCs, and aldehydes in 2050. The specificity of this model is that it considers changes in housing stocks, including the construction of millions of houses in the US over the 2010–2050 period, the fact that some existing houses will be renovated or demolished, and the population demography changes. The home construction and renovation are modeled as a change in their envelope, i.e., higher airtightness, and heating/cooling systems. The model takes into consideration infiltration, ventilation, deposition, reaction, and pollutant removal by HVAC filters. The results showed that the indoor annual mean concentrations of PM_2.5_, UFP, and NO_2_ would decrease due to the drop from both indoor (substitution of gas stoves by electric stoves) and outdoor sources (less infiltration and window opening). The indoor concentration of ozone originated outdoors would increase, thus promoting indoor chemical reactions. The indoor annual mean concentrations of formaldehyde, acetaldehyde, acrolein, 1,3-butadiene, benzene, and p-dichlorobenzene would increase due to the decrease in infiltration and duration of open windows.

**Table 1:** Models developed for the assessment of the influence of climate change on indoor air quality. (Note: The definitions of the variables are provided in the nomenclature.).

References	Notes
Chang et al. [10]	Equation: $V\frac{d\left[C_{V}\right]}{\mathrm{dt}}=\left(Q_{\mathrm{mv}}C_{V_{0}}+Q_{\mathrm{ra}}C_{V}\right)\left(1-e\right)+(Q_{\mathrm{cf}}+Q_{\mathrm{window}})C_{V_{0}}+Q_{{\mathrm{ADJ}}_{0}}C_{V_{{\mathrm{ADJ}}_{0}}}-\left[\left(Q_{\mathrm{mv}}+Q_{\mathrm{ra}}+Q_{\mathrm{cf}}+Q_{{\mathrm{ADJ}}_{0}}+Q_{\mathrm{window}}\right)C_{V}\right]-\left(\sum_{i}E_{s,i}A_{i}+{\mathrm{Vk}}_{V}C_{V}+Q_{\mathrm{rcl}}e_{\mathrm{rcl}}C_{V}\right)+\sum_{j}E_{V,j}$; in the gas phase.$V\frac{d\left[M\left(d\right)C_{p}\left(d\right)\right]}{\mathrm{dt}}=\left[Q_{\mathrm{mv}}M_{0}\left(d\right)C_{p_{0}}\left(d\right)+Q_{\mathrm{ra}}M\left(d\right)C_{p}\left(d\right)\right]\left[1-e\left(d\right)\right]+Q_{\mathrm{cf}}M_{0}\left(d\right)C_{p_{0}}\left(d\right)P+Q_{\mathrm{window}}M_{0}\left(d\right)C_{p_{0}}\left(d\right)+Q_{{\mathrm{ADJ}}_{0}}M_{{\mathrm{ADJ}}_{0}}\left(d\right)C_{P_{{\mathrm{ADJ}}_{0}}}\left(d\right)-\left(Q_{\mathrm{mv}}+Q_{\mathrm{ra}}+Q_{\mathrm{cf}}+Q_{{\mathrm{ADJ}}_{0}}+Q_{\mathrm{window}}\right)M\left(d\right)C_{p}\left(d\right)-\left[\sum_{i}A_{i}v_{\mathrm{dep}}M\left(d\right)C_{p}\left(d\right)+Q_{\mathrm{rcl}}e_{\mathrm{rcl}}\left(d\right)M\left(d\right)C_{p}\left(d\right)\right]+\left[\sum_{k}M_{\mathrm{pk}}\left(d\right)C_{\mathrm{pk}}\left(d\right)+R_{\mathrm{sus}}M_{f}\left(d\right)C_{f}\left(d\right)A_{\mathrm{dtot}}\right]$; in the PM phase. Scope of application: VOCs and SVOCs. Main features: Two-compartment dynamic model, suggesting that the effect of temperature on mass transfer parameters, pollutant emission from materials, and window opening behavior are the key factors to be considered in the context of climate change.
Ilacqua et al. [11]	Equation: $C_{\mathrm{in}}=\frac{P\lambda }{\lambda +d_{s}}C_{\mathrm{out}}+\frac{S}{V\left(\lambda +d_{s}\right)}$ Scope of application: radon, PM_2.5_, carbonyls, ozone, NO_2_, HNO_3_, UFP Main features: Single-compartment steady-state model considering infiltration as the only airflow path from outdoors to indoors. The model also considers the emission from indoor sources. However, it does not consider possible changes in the airtightness of the building envelopes and the interactions between vapor and solid phases (particles and surfaces).
Salthammer et al. [12]	Equation: $\frac{{\mathrm{dC}}_{\mathrm{in}}}{\mathrm{dt}}={\lambda C}_{\mathrm{out}}-{\lambda C}_{\mathrm{in}}-\lambda_{d}C_{\mathrm{in}}$ for ozone $\frac{{\mathrm{dC}}_{\mathrm{in}}}{\mathrm{dt}}={\lambda \mathrm{PC}}_{\mathrm{out}}-{\lambda C}_{\mathrm{in}}-\lambda_{d}C_{\mathrm{in}}$ for particles Scope of application: ozone, particles Main features: Single-compartment dynamic model. The model does not consider the evolution of the building envelope, window opening, or mechanical ventilation systems and the emissions from indoor sources.
Salthammer et al. [14]	Equation: $\frac{{\mathrm{dC}}_{\mathrm{in}}}{\mathrm{dt}}={P\lambda C}_{\mathrm{out}}-C_{\mathrm{in}}+\sum_{i=1}^{n}\frac{E_{s,i}A_{i}}{V}+\sum_{j=1}^{n}\frac{E_{V,j}}{V}\pm J_{\mathrm{coag}}\pm {\xi \Psi }_{\mathrm{gas}}\pm J_{\mathrm{SVOC}}$ Scope of application: VOCs and SVOCs Main features: Two-compartment dynamic model, combining 5 submodels to assess the building physics, emissions from indoor sources, reactions, particle/gas partitioning, mold growth, and human exposure.
Fazli and Stephens [15] Fazli et al. [16]	Equation: $\frac{{\mathrm{dC}}_{\mathrm{in}}}{\mathrm{dt}}=\left(\left({P\lambda }_{\inf}+\lambda_{\mathrm{nat}}\right)C_{\mathrm{out}}+\frac{s}{V}-(\lambda_{\inf}+\lambda_{\mathrm{nat}}\right))C_{\mathrm{in}}-\frac{Q_{\mathrm{exhaust}}}{V}C_{\mathrm{in}}-{\beta C}_{\mathrm{in}}-f_{\mathrm{filt}}\frac{\eta_{\mathrm{filt}}Q_{\mathrm{filt}}}{V}C_{\mathrm{in}}-{\mathrm{kC}}_{\mathrm{terp}}C_{\mathrm{in}}$ Scope of application: PM_2.5_, UFP, NO_2_, ozone, VOCs, aldehydes Main features: Single-compartment dynamic model, that considers changes in building envelope airtightness, use of HVAC systems, and population demography changes.

### 3.2. Pollutant-Related Factors

#### 3.2.1. Effect of Indoor Temperature and Humidity on Pollutant Emissions, Transport, and Partitioning Indoors

One of the effects of climate change is the increase in temperature and relative humidity outdoors and consequently indoors, with more frequent overheating periods. Hence, it is important to characterize the effect of temperature and relative humidity on the pollutant emission from indoor materials, as well as on other pollutant transport processes. Fang et al. [17] studied experimentally the impact of indoor temperature and humidity on the emission of total volatile organic compounds (TVOCs) from 5 types of building materials: PVC (polyvinyl chloride) flooring, loomed polyamide carpet, acrylic sealant, acrylic wall paint, and waterborne acrylic floor varnish. The emissions were measured for 9 combinations of temperature (18, 23, and 28 °C) and humidity (30, 50, and 70%). The results showed that for the wall paint and floor varnish, the one-week mean TVOC concentrations increased significantly by 1500 µg·m^−3^ when relative humidity increased from 30 to 70%; the one-week mean TVOC concentration emitted from the floor varnish also increased by 700 µg·m^−3^ when temperature increased from 18 to 28 °C. For the carpet, PVC flooring, and sealant, no clear impact of temperature and humidity on the emission of TVOCs was observed. Similar results on the same types of materials were reported by Wolkoff [18]: the effect of temperature and relative humidity is dependent on the building materials/products and the VOCs emitted. Based on 3675 air samples collected in two locations in China for 23 months, the combined effect of indoor temperature and humidity on indoor VOC concentrations was addressed in the model of Zhou et al. [19], where the estimated daily average concentration of a VOC (mg·m^−3^) in newly renovated residences is calculated as follows:

(1) $$C=k_{1}e^{n_{1}T}\;k_{2}e^{n_{2}RH}+C_{0}$$

where *T* (°C) and *RH* (%) are the room’s daily average temperature and the relative humidity, respectively. *k*_1_, *k*_2_, *n*_1_, and *n*_2_ are constants related to the pollutant and other environmental conditions. *C*_0_ (mg·m^−3^) is the concentration under initial temperature and relative humidity. As the equation indicates, the indoor VOC concentration is exponentially correlated with temperature and relative humidity. Zhou et al. [19] also showed that k_1_, k_2_, n_1_, or n_2_ were correlated with Henry’s law constants (solubility in water) and molecule polarity for formaldehyde, butyl acetate, styrene, benzene, toluene, xylene, ethylbenzene, and n-undecane. Nevertheless, the equation has not been validated for long periods, e.g., for decades.

Xiong et al. [20] presented another model describing the combined effect of indoor temperature and relative humidity on emissions of formaldehyde and VOCs from building materials. The equation, theoretically developed from some physical and chemical equations, can be written as follows:

(2) $$E=E_{1}T^{0.75}e^{E_{2}RH}-\frac{E_{3}}{T}$$

where *E* is the steady-state emission rate (µg·m^−2^·h^−1^); *E*_1_, *E*_2_, and *E*_3_ are positive constants related to the physical and chemical characteristics of the pollutant; *T* (K) and *RH* (%) are the indoor temperature and the relative humidity, respectively. This correlation was validated using experimental data from fiberboard and floor varnish emissions under different combinations of temperature and relative humidity. The constants were determined with an acceptable fitting degree. 

The effect of temperature on VOC emissions is a widely discussed topic. Salthammer and Morrison [21] listed 36 articles, published between 1975 and 2021, on the temperature dependence of VOC emissions from different building materials. The review of the 36 articles covered various families of pollutants and compiled different methods to study the dependence of VOC emissions on indoor temperature. 

The diffusion coefficient (diffusivity) of a pollutant from a surface to the air is an important factor primarily used to calculate the mass transfer coefficient. Guo [22] summarized 3 methods for the estimation of the diffusivity of VOCs in air, among them two are directly dependent on the temperature and are presented in the following equations:

(3) $$D=\frac{10^{-3}T^{1.75}\sqrt{M_{r}}}{P_{0}{\left(v_{A}^{\frac{1}{3}}+v_{B}^{\frac{1}{3}}\right)}^{2}}$$

(4) $$D=\frac{BT^{\frac{2}{3}}\sqrt{M_{r}}}{P_{0}\sigma_{AB}^{2}\gamma }$$

where *D* is the diffusivity (cm^2^·s^−1^), *T* is the temperature (K), *P*_0_ is the atmospheric pressure (atm), *v*_A_ and *v*_B_ are the molar volumes for air and the studied compound (cm^3^·mol^−1^), *σ*_AB_ is the characteristic length (Å), *γ* is the collision integral for diffusion (dimensionless).

In Equations (3) and (4) M_r_ and *B* are defined as:

$M_{r}=\frac{M_{a}+M}{M_{a}M}$, *M*_a_, and *M* (mol·g^−1^) are the molecular weights of air and the studied compound.

$$B=0.0217-0.0005M_{r}^{\frac{1}{2}}$$

The effect of temperature on the diffusion coefficient is quantified by applying the previous two equations. The results are shown in Table 2. A temperature increase of 10 °C can increase the diffusion coefficient by around 6%. This variation should not be neglected when calculating the mass transfer coefficient because it could have an important effect on the pollutant concentration in the gas phase.

Wei et al. [23] derived theoretically the equations of the particle/gas partition coefficient *K*_p_ of some SVOCs as a function of temperature. Table 3 shows the values of *K*_p_ of some phthalates at 20 °C and the percentage of variation of *K*_p_ when the temperature rises from 20 °C to 30 °C. This percentage may reach 78%, which is an important variation for a realistic and possible temperature increase.

Fadeyi [24] showed that ozone surface deposition velocity increased when relative humidity and temperature increased. For some materials such as concrete, the increase in ozone deposition velocity depended on the range of relative humidity; it appeared more clearly at humidity higher than 50% [24]. The ozone deposition velocity can increase by a factor of 17 when humidity increased from 50% to 90%, depending on the material type [25]. 

Salthammer and Morrison [21] highlighted the effect of temperature on the reaction rates of some pollutants with oxidants. They provided eight examples of indoor gas-phase reactions between ozone and hydroxyl as oxidants and nitrogen monoxide (NO), NO_2_, limonene, α-pinene, and β-pinene, along with their first-order reaction rates at 25 °C and the percentages of variation of these rates when the temperature increased to 35 °C: the variation ranged from −5 to 30.6% for these eight reactions.

Beyond their own interest in the characterization of the impact of thermal conditions on pollutant emission, transport and partitioning, these relationships, when integrated into an IAQ model, will allow us to characterize the evolution of indoor pollutant concentrations following the evolution of indoor thermal conditions.

#### 3.2.2. Outdoor Concentration Evolution in the Future Climate Conditions

Outdoor air quality is an important determinant of IAQ since outdoor air is transferred into the building through ventilation and infiltration. Therefore, it is necessary to know how outdoor pollutant concentration will evolve under climate change conditions to anticipate the impact on IAQ.

Ozone is produced essentially outdoors [26] in the presence of ultraviolet light and precursors such as NO_x_ [27]. High concentrations of ozone are recorded during heat waves [28] because high temperatures promote its formation [29]. Wang et al. [30] showed that, over East China, climate change alone, i.e., excluding the evolution of anthropogenic emissions, might be responsible for 8% of the total increase in annual-mean surface ozone until 2050. The same study [30] showed that in West China ozone concentration might decrease by 4% due to climate change. This ozone concentration difference between East and West China is because the West China region is less-industrialized and is therefore a low-NO_x_ area [30]. The study by Hong et al. [31] in China showed that ozone concentration will increase in the urban regions in 2046–2050 by 16 µg·m^−3^, compared to the 2006–2010 years. In Europe, Meleux et al. [32] predicted the increase in ozone concentration under climate change conditions, i.e., with increased temperature and decreased cloudiness, considering current anthropogenic emissions, especially for the western and central regions. The number of days of 90 and 120 ppb ozone exceedance will double or triple in some European cities depending on the climate scenario [32]. Coelho et al. [33] predicted a decrease in ozone concentration (up to 30 µg·m^−3^) over Europe in 2031, compared to 2013, considering the effect of climate change and assuming constant anthropogenic emissions. Zhong et al. [34] studied the impact of climate change on ozone production from VOCs, methane, and carbon monoxide outdoors, and showed the effect of high temperatures on ozone formation. A higher temperature fosters photochemical reactions, VOC emission from vegetation, and evaporation, suggesting more humidity and OH radicals in the atmosphere, which leads to a higher outdoor ozone concentration [34]. 

By applying two climate models, Coelho et al. [33] predicted an increase in the annual mean NO_2_ concentrations (up to 5 µg·m^−3^) in some European regions and a decrease in other European regions (up to 5 µg·m^−3^). Globally, atmospheric NO_2_ concentrations are expected to increase in the 21st century despite the decreased cloudiness due to the increasing boundary layer height [33]. Giorgi and Meleux [35] showed that the NO_x_ concentration should increase in most European regions in the 2071–2100 period by 0–1 ppb compared to the 1961–1990 period, under the high CO_2_ emission scenario, due to lower mixing and dispersion over Europe and lower total deposition specifically in the capital cities, such as Paris, London, and Brussels. 

Coelho et al. [33] predicted an increase in PM_2.5_ and PM_10_ concentrations (up to 30 µg·m^−3^) in Europe. In China, PM_2.5_ concentration will increase in the urban regions in 2046–2050 by 8 µg·m^−3^ compared to the 2006–2010 years [31]. Particle concentration is expected to increase under the effect of the lower precipitation that reduces dispersion, dilution, and wet deposition of particles. Westervelt et al. [36] used a multiple linear regression (MLR) model to characterize the correlation between meteorological parameters and atmospheric PM_2.5_ concentrations. The temperature and PM_2.5_ concentrations were positively and significantly correlated. PM_2.5_ concentration presented a negative correlation with wind speed due to the dilution effect. Other meteorological conditions such as precipitation, cloudiness, pressure, and relative humidity showed a negative poor correlation with PM_2.5_ concentrations in most regions of the world. The decrease in heating demand due to warmer winters and the decrease in fossil fuel combustion could counterbalance the increase in PM_2.5_ outdoor concentrations [9]. In addition, outdoor air pollution control regulation, reduction of sulfur dioxide emissions from coal-fired power plants due to climate change concerns, and the shift toward electric and hybrid vehicles could reduce particulate matter in outdoor air. On the other hand, outdoor particulate matter concentrations may also increase as a result of more frequent drought episodes leading to more windblown dust and wildfires [3].

Giorgi and Meleux [35] showed that global warming might lead to a higher concentration of isoprene (a biogenic VOC) in France, by 0–8 ppb from 1961–1990 to 2071–2100 period. The modeling results of Cao et al. [37] on isoprene showed an increasing global emission in the 21st century caused by a warming climate. The annual total isoprene emissions will rise by 100 to 250 million tons of carbon in 2100 compared to the 1850–1950 years. 

The trends in the evolution of outdoor pollutant concentrations are summarized in Table 4. Overall, differences in the tendencies of variation of most of the outdoor concentrations can be noticed depending on the geographical zone, and the scenarios considered for their prediction.

### 3.3. Building-Related Factors

#### 3.3.1. Climate Change Mitigation and Adaptation Actions

Some modifications in the insulation and construction materials can be made to mitigate the effect of climate change and adapt buildings. Substitution of concrete with wood in construction is a way to mitigate climate change by reducing carbon emissions [38,39]. However, the weakness of wood compared to concrete in terms of resistance to high humidity and fungi is still a major limitation of its use in construction [40]. Furthermore, wood products emit formaldehyde, terpenes, and BTEX (benzene, toluene, ethylbenzene, and xylene) [41,42]. The use of natural and bio-based insulating materials, e.g., cellulose, cork, straw bale, wood wool, and sheep wool, in insulation, has taken off in the last few years, besides the appearance of new materials [43]. Although straw bale houses are not VOC-free, they show lower VOC indoor concentrations than regular houses [44]. The integration of new insulation materials in buildings can impact indoor thermal conditions, and thus air infiltration and emissions of pollutants from indoor sources. Verichev et al. [45] presented the results of the energy simulation of an existing house in Chile and calculated the cost-effective optimum thickness of insulation materials for the period of 2020–2035. They found that the geographical area of usage of glass wool as insulation material can be doubled in 2020–2035 in comparison with 2006 in Chile. The different insulation materials do not emit the same quantity of pollutants, which can vary by two orders of magnitude [46,47]. 

The buildings will also evolve to counteract the high temperature indoors. Installation of shading devices could neutralize a proportion of the energy required for cooling in future years [48]. Techniques to mitigate the intensity of urban heat islands (UHI) and reduce both outdoor and indoor temperatures are also developed [49]. For instance, the use of green technologies, e.g., urban green spaces, green roofs, and green walls, can provide a mitigation potential of outdoor temperature in the range of 0.3–2.5 °C [49]. This range can be greater when these green technologies are mixed with other techniques such as the use of reflective materials and water technologies. A profound change in the built environment is foreseen, for which the influence on IAQ remains unknown. 

#### 3.3.2. Effect of Outdoor Temperature on Air Infiltration

In many countries, building regulations require airtight envelopes to prevent heat loss by infiltration and improve the effectiveness of mechanical ventilation systems [2]. This increase in building airtightness might degrade IAQ if mechanical ventilation is not properly installed or maintained [50], but it may also lower pollutant transport from outdoors to indoors. 

Lee et al. [51] studied the PM_2.5_ infiltration, aiming to quantify the relationship between future changes in outdoor temperature and fine particle infiltration in the Greater Boston area. The study used the indoor-outdoor sulfur ratio as an indicator of PM_2.5_ infiltration due to the absence of indoor sulfur sources. An increase in outdoor temperature of 2–3 °C in summer corresponds to up to 0.06 increase in the indoor-outdoor sulfur ratio. A similar result was obtained by Ilacqua et al. [11], who suggested that infiltration rates could increase by up to 25% in the summer months in the 2040–2070 period, compared to the reference period (1970–2000), assuming a constant indoor temperature of 24.9 °C. These infiltration rates were calculated using an equation from the Lawrence Berkeley National Laboratory model, taking into consideration the indoor-outdoor temperature difference and the wind effect [11]. However, based on their residential energy and indoor air quality model, Fazli et al. [16] found that infiltration factors (“the equilibrium proportion of particles remaining suspended on penetrating indoors” [52]) of PM_2.5_, UFP, NO_2_, and ozone in 2050 would be similar to those in 2010 because the effects of climate and building stoke changes neutralize each other. On the other hand, indoor-outdoor concentration ratios will be higher in 2050 for PM_2.5_, ultrafine particles, and NO_2_ in homes equipped with gas stoves compared to the same ratios in 2010 due to a lower natural ventilation rate.

### 3.4. Human-Related Factors

Human activities (e.g., cooking, smoking, etc.) and behavior (e.g., window opening) can significantly influence IAQ [53]. Changes in window opening can modify the air change rate and airflow velocities at indoor surfaces, which directly affect indoor pollutant concentrations. Under future climate conditions, window opening could increase from autumn to spring because of warmer weather and decrease in summer to protect from heat waves. Moreover, it is expected that the use of cooling systems will increase; such an increase has already been observed in the past years all over the world [54,55]. With the widespread use of cooling systems, the duration of open windows could drastically decrease. Predicting IAQ in the context of climate change requires being able to model window opening considering these different aspects. 

According to Huang et al. [56], the air change rate is the most influencing factor on VOC emission rates from source materials: it is associated with the emission rates of 16 VOCs among 43 VOCs studied, while temperature and relative humidity are associated with 7 and 6 VOCs, respectively. The materials in the test residences were composite and solid wood for flooring, and paints and wood boards for walls. Window opening may become less effective in terms of thermal comfort under climate change conditions and other ventilation and air conditioning strategies may substitute it or become predominant [57]. 

Window opening is often modeled using stochastic/probabilistic models. For example, Rijal et al. [58] developed a stochastic model to predict occupant behavior in Japanese dwellings, considering cases of heated, cooled, and free-running dwellings. The model relates the probability of windows being open to indoor and outdoor temperatures based on adaptive thermal comfort. Andersen et al. suggested including other indoor parameters, such as indoor illuminance and wind speed, for the determination of window opening probability [59], and a multivariate regression method was proposed in the following form:

(5) $$\log\frac{p}{1-p}=a+b_{1}x_{1}+b_{2}x_{2}+\ldots +b_{n}x_{n}+c_{12}x_{1}x_{2}+c_{13}x_{1}x_{3}+\ldots$$

where *p* is the probability of window opening/closing, *a* and *b*_1-n_ are empirical coefficients, and *x*_1-n_ stand for the driving factors for windows changing state. The investigated variables included indoor and outdoor temperature, indoor and outdoor relative humidity, indoor illuminance, wind speed, solar radiation, and sunshine hours. Equation (5) was further simplified by ignoring the interactions between variables. Andersen et al. [59] used the simplified regression equation, identified the most influential variables, and provided their coefficients based on measurements conducted in 15 houses in Denmark in 2008. These stochastic/probabilistic models were commonly developed under current climate conditions, although some have considered climate change. 

Liu et al. [60] applied a statistical method to calculate the variation of the time fraction of open windows in China in 2050 compared to that in 2015. The results showed a decreasing time fraction in summer by up to −5% depending on the climate scenario due to the rising use of air conditioning systems, and an increasing time fraction by up to 2.8% in winter because of a warmer outdoor climate.

Chang et al. [10] considered in their model that the outdoor temperature controls the heating and cooling system to maintain a comfortable temperature indoors. The heating and cooling system was turned on if the outdoor temperature was below 10 °C or above 30 °C, and windows were closed. Between 10 °C and 30 °C outdoors, the indoor temperature was mainly controlled through window opening. The application of this algorithm to the 2071–2100 climate conditions in South Korea resulted in a increase in window opening duration by 200% in winter, 50% in spring, and 20% in autumn compared to the 1976–2005 years. However, window opening duration dropped by up to 55% in summer. A study conducted in China by Du et al. [61] on 10 houses with different characteristics (area, year of construction, occupancy, floor, etc.) showed that the window opening probability by human control was governed by the daily and yearly periods, the indoor-outdoor temperature difference and the occupant perception. Other determinants of window opening, with different degrees of dependence on climate change, such as wind speed, solar radiation, age and gender of occupants, smoking activities, orientation, and size of windows, were described in detail by Fabi et al. [62]. In summary, all the investigated articles suggest that the duration of window opening will decrease in summer and increase in other seasons. Consequently, the reduced ventilation rate will increase exposure to radon and pollutants emitted from indoor sources such as materials, occupants, and combustions [3].

## 4. Conclusions

The existing studies provide knowledge about most of the phenomena associated with climate change and the way they can influence indoor air quality. However, it is still difficult to get a quantitative evaluation of the combined and possibly antagonistic effects of climate change on indoor pollutant concentrations. Modeling is a suitable method to achieve this goal, and the literature review shows that IAQ modeling coupled with heat and airflow modeling is needed. Across the IAQ models that have been developed and used to predict indoor pollutant concentrations, those that are the most advanced in terms of modeling all physical and chemical processes on a wide range of pollutants, are not yet applied to future climate scenarios. Moreover, parameters have been disregarded in some models without prior analysis of their sensitivity to the output, which brings conclusions to caution.

Measurements of VOC emissions from materials suggest that the VOC emission rate can increase significantly under increased temperature, thus global warming and heat waves in summer may lead to higher indoor VOC source emissions in the years to come. Most studies described only qualitatively the impact of environmental, building, and human factors on indoor pollutant concentrations. Empirical laws and stochastic models of window opening were developed and could be used for further in-depth analysis of the influence of ventilation on IAQ in the context of climate change.

In summary, the influence of climate change on IAQ remains largely unknown and the evolution of many influencing factors is unpredictable, such as the technological development in the formulation and manufacturing of building materials or the evolution of building codes and building stocks in the medium term (2050) and the long term (2100). This influence is likely to vary across the countries because of the differences in building, socio-economic and cultural characteristics, public policies, and the local evolution of the climate.

## Figures and Tables

**Table 2:** Effect of temperature on the diffusion coefficient.

Change in Temperature (°C)	Increase in Diffusion Coefficient (%)	
	Equation (3)	Equation (4)
15 to 25	6.2	2.3
25 to 35	6	2.2
35 to 45	5.8	2.2

**Table 3:** Variation of *K*_p_ of some phthalates with changing temperature.

Phthalate	*K_p_* at 20 °C (m^3^·µg^−1^)	Variation (%) When *T* Rises to 30 °C
DEHP	4.3 × 10^−2^	−74
DnBP	3 × 10^−4^	−71
DiBP	2 × 10^−4^	−71
DiNP	34.082	−78
BBzP	8 × 10^−4^	−69
DEP	7.6 × 10^−6^	−68
DMP	3.3 × 10^−6^	−62

**Table 4:** Predicted trends in outdoor pollutant concentrations under future climate conditions. ↑: increase, ↓: decrease, ↑ ↓: increase in some geographical zones and decrease in others.

Pollutant	References	Tendency	Region
Ozone	Wang et al. [30]	↑ ↓	China
	Hong et al. [31]	↑	China
	Meleux et al. [32]	↑	Europe
	Coelho et al. [33]	↓	Europe
	Giorgi et Meleux [35]	↑	Europe
NO_2_	Coelho et al. [33]	↑ ↓	Europe
NO_x_	Giorgi et Meleux [35]	↑	Europe
Particles (PM_2.5_ and PM_10_)	Coelho et al. [33]	↑	Europe
PM_2.5_	Hong et al. [31]	↑	China
Isoprene	Giorgi et Meleux [35]	↑	Europe
	Cao et al. [37]	↑	World

## Nomenclature

A_dtot_	The total area of the downward-facing interior surface (m^2^)
A_i_	Area of interior surface *i* (m^2^)
C_f_(d)	Concentration associated with M_f_(d) (µg·µg^−1^ Particulate matter)
C_in_	Indoor concentration (µg·m^−3^)
C_out_	Outdoor concentration (µg·m^−3^)
C_p_ (d)	Mass concentration sorbed to particulate matter (PM) in the air compartment (µg·µg^−1^ PM)
C_p0_ (d)	Mass concentration sorbed to PM in the outdoor air (µg·µg^−1^ PM)
C_pADJ0_ (d)	Mass concentration sorbed to PM in the adjoining room (µg·µg^−1^ PM)
C_Pk_(d)	Concentration associated with M_Pk_(d)
C_terp_	Concentration of a reactant (µg·m^−3^)
C_V_	Concentration in the vapor phase of the air compartment (µg·m^−3^)
C_V0_	Concentration in the vapor phase of the outdoor air (µg·m^−3^)
C_VADJ0_	Concentration in the vapor phase of the adjoining room (µg·m^−3^)
d	Aerodynamic diameter of PM (µm)
d_s_	First-order decay rate constant combining all sink processes (h^−1^)
e	Filter efficiency of the mechanical ventilation system (dimensionless)
e(d)	Filter efficiency of the mechanical ventilation system of PM (dimensionless)
e_rcl_	Efficiency of the indoor filter device (dimensionless)
e_rcl_ (d)	Filter efficiency of the indoor recirculation device of PM (dimensionless)
E_si_	Emission rate from the interior surface i (µg·h^−1^·m^−2^)
E_Vj_	Emission rate from indoor source *j* other than interior surfaces (µg·h^−1^)
f_filt_	Fractional runtime of the HVAC system if applicable (dimensionless, ranging from 0 to 1)
J_coag_	Coagulation term for gain or loss of the target size particle (µg·m^−3^·h^−1^)
J_SVOC_	SVOC loss as gas phase and gain as particle phase (µg·m^−3^·h^−1^)
k	Bimolecular reaction rate constant between two gas-phase compounds(m^3^·µg^−1^·h^−1^)
K_V_	First-order reaction rate constant in the gas phase (h^−1^)
M (d)	PMs mass concentration in the air compartment (µg PM·m^−3^)
M_0_ (d)	PMs mass concentration in the outdoor air (µg PM·m^−3^)
M_ADJ0_ (d)	PMs mass concentration in the air of the adjoining room (µg PM·m^−3^)
M_f_(d)	PMs concentration on the floor surface (µg PM·m^−2^)
M_Pk_(d)	PMs emission rate from indoor source k (µg PM·h^−1^)
P	Penetration factor through cracks (dimensionless, often close to 1)
Q_ADJ0_	Volumetric flow rate from the adjoining room (m^3^·h^−1^)
Q_cf_	Volumetric flow rate of air through cracks (m^3^·h^−1^)
Q_exhaust_	Airflow rate of any mechanical exhaust ventilation system (m^3^·h^−1^)
Q_filt_	Airflow rate through the central HVAC filter if applicable (m^3^·h^−1^)
Q_mv_	Volumetric flow rate of air drawn to the mechanical ventilation system (m^3^·h^−1^)
Q_ra_	Volumetric flow rate of return air to the mechanical ventilation system (m^3^·h^−1^)
Q_rcl_	Air recirculation rate of the indoor filter device (m^3^·h^−1^)
Q_window_	Natural airflow through the window opening (m^3^·h^−1^)
R_sus_	Resuspension rate coefficient from surface to indoor air (h^−1^)
S	Indoor source strength (mass emission rate) (µg·h^−1^)
t	Time (h)
V	Volume of the compartment (m^3^)
v_dep_	Deposition velocity from indoor air to surface (m·h^−1^)
β	The first-order indoor loss rate by deposition to surfaces and/or surface reactions (h^−1^)
η_filt_	Removal efficiency of a filter installed in the HVAC system if applicable (dimensionless, ranging from 0 to 1)
ξ	Secondary organic aerosols yield
Ψ_gas_	The production or removal rate of a species due to gas-phase reaction (µg·m^−3^·h^−1^)
λ	Air exchange rate (h^−1^)
λ_d_	Deposition rate of particles or gas species on indoor surfaces (h^−1^)
λ_inf_	Air exchange rate due to infiltration alone (h^−1^)
λ_nat_	Air exchange rate due to the natural ventilation (h^−1^)
